# TNF-α increases breast cancer stem-like cells through up-regulating TAZ expression via the non-canonical NF-κB pathway

**DOI:** 10.1038/s41598-020-58642-y

**Published:** 2020-02-04

**Authors:** Wenjing Liu, Xiaoqing Lu, Peiguo Shi, Guangxi Yang, Zhongmei Zhou, Wei Li, Xiaoyun Mao, Dewei Jiang, Ceshi Chen

**Affiliations:** 1 Key Laboratory of Animal Models and Human Disease Mechanisms of the Chinese Academy of Sciences and Yunnan Province, Kunming Institute of Zoology, Chinese Academy of Sciences, Kunming, 650223 China; 2 University of the Chinese Academy of Sciences, Beijing, 101407 China; 3 Medical Faculty of Kunming University of Science and Technology, Kunming, Yunnan 650500 China; 4 Department of breast surgery, The second hospital of Shanxi medical University, Taiyuan, 030071 China; 5 Department of Urology of the First People’s Hospital of Yunnan Province, Kunming, 650032 China; 6 Breast surgery, The First Affiliated Hospital of China Medical University, Shenyang, 110001 China; 7 KIZ-CUHK Joint Laboratory of Bioresources and Molecular Research in Common Diseases, Kunming Institute of Zoology, Chinese Academy of Sciences, Kunming, 650223 China

**Keywords:** Cancer stem cells, Inflammation, Breast cancer

## Abstract

Breast cancer patients often suffer from disease relapse and metastasis due to the presence of breast cancer stem-like cells (BCSCs). Numerous studies have reported that high levels of inflammatory factors, including tumor necrosis factor alpha (TNF-α), promote BCSCs. However, the mechanism by which TNF-α promotes BCSCs is unclear. In this study, we demonstrate that TNF-α up-regulates TAZ, a transcriptional co-activator promoting BCSC self-renewal capacity in human breast cancer cell lines. Depletion of TAZ abrogated the increase in BCSCs mediated by TNF-α. TAZ is induced by TNF-α through the non-canonical NF-κB pathway, and our findings suggest that TAZ plays a crucial role in inflammatory factor–promoted breast cancer stemness and could serve as a promising therapeutic target.

## Introduction

Breast cancer is one of the most common malignances and a serious threat to women’s health worldwide^1^. Inflammation, especially chronic inflammation, plays an important role in cancer initiation and progression^2^. Tumor cells and a variety of leukocytes attracted by tumor cells produce various cytokines and chemokines that affect cancer development^3^. In general, cytokines are divided into two groups. One group comprises pro-inflammatory factors, including tumor necrosis factor alpha (TNF-α), IL1β, IL-6, etc^4,5^. The other group is made of anti-inflammatory factors, including IL-10, IL-13, etc^5^. High levels of pro-inflammatory cytokines promote tumor growth and migration, enhance the survival of malignant cells, suppress adaptive immune responses, and cause resistance to hormones and chemotherapeutic agents^6,7^. Non-steroidal anti-inflammatory drugs decrease the risk for developing and the risk of mortality in breast cancer^3^. Characterization of the mechanisms by which inflammatory cytokines promote breast cancer development may offer new therapeutic opportunities.

TNF-α is a well-documented pro-inflammatory cytokine that is up-regulated in breast cancer, and high levels of TNF-α are associated with breast cancer recurrence^8,9^. Additionally, TNF-α levels are positively correlated with tumor grade in serous ovarian tumors^10^. Moreover, TNF-α knockout mice are less susceptible to DMBA- or TPA-induced skin tumors^8^. TNF-α binds to two different receptors, TNF-α receptor 1 and 2 (TNFR1/2), to activate the NF-κB signaling pathway^11,12^. TNFR1/2 activates IKK and subsequently causes IκBα phosphorylation, ubiquitination, and degradation, leading to p65, RelB or p50 translocation to the nucleus. In addition to the canonical NF-κB pathway, TNF-α is able to activate JNK, MAPKs, AKT, and the non-canonical NF-κB pathway^13–15^. TNF-α up-regulates over 400 inflammatory genes, including cell-adhesion molecules, anti-apoptotic proteins, inflammatory cytokines, and chemokines^16,17^.

Ginalu Storci *et al*. reported that TNF-α increases the proportion of breast cancer stem-like cells (BCSCs) through NF-κB/HIF1α/Slug^18^. BCSCs are a small subpopulation of the primary breast tumor with differentiation and self-renewal capacities that are resistant to chemo- and radio-therapies^19,20^. Aldehyde dehydrogenases positivity and CD44^high^ CD24^low^ are generally considered two of the most frequently used identification makers of BCSCs^21^. It has been reported that CD44 positive/high expression is responsible for maintenance of multipotency, proliferation, and migration^22^, CD24 negative/low expression is responsible for cell growth and migration^23^, and ALDH1 positive/high expression is responsible for cell proliferation and stemness^24^. In addition, mammosphere formation can also identify breast cancer stem-like cells, which is based on the ability of BCSCs to propagate as multicellular spheroids in suspension culture. Currently, BCSCs have been implicated in breast cancer relapse and metastasis due to their resistance to chemo- and radiotherapies and tumorigenic properties^25,26^. However, there are currently no effective therapeutic strategies to specifically eliminate BCSCs.

TAZ, a transcriptional co-activator with a PDZ binding motif, has been implicated in sustaining BCSCs. A previous study demonstrated that TAZ confers self-renewal and tumor initiation capacity to non-BCSCs^27^. As a main effector of the Hippo pathway, TAZ interacts with TEADs to activate transcription of target genes, including *CYR61*, *CTGF*, and *BIRC5*^28–30^. High TAZ expression is associated with low survival rates in breast cancer patients^27^. Our previous study suggested that TAZ depletion dramatically suppresses basal type breast cancer HCC1937 growth *in vivo*^31^. While regulation of TAZ at the post-translational modification level is becoming increasingly clear, knowledge of TAZ regulation at the transcriptional level is relatively limited. To date, four transcription factors, RelA^32^, HIF-1^33^, SRF and MRTF^34^, have been reported to promote TAZ transcription.

Herein, we illustrate that TNF-α increases the percentage of BCSCs and TAZ expression levels in human breast cancer cell lines, and depletion of TAZ abrogates this phenotype. We further demonstrate that TAZ is induced by TNF-α through the non-canonical NF-κB pathway. Our findings indicate that inflammatory factors such as TNF-α increases stemness via up-regulation of TAZ transcription through non-canonical NF-κB pathway. We suggest that TAZ plays a crucial role in TNF-α–promoted breast cancer stemness and could serve as a promising therapeutic target.

## Results

### TNF-α increases breast cancer stem-like cells and up-regulates TAZ transcription in breast cancer cell lines

Two breast cancer cell lines, MCF7 and MDA-MB-468, were used in this study. MCF7 is ERα+ breast cancer cell line and MDA-MB-468 is triple-negative breast cancer (TNBC) cell line. It has been reported that TNBC cells are more like cancer stem cells (CSC) in terms of gene expression signature^35^.

**Figure 1:**
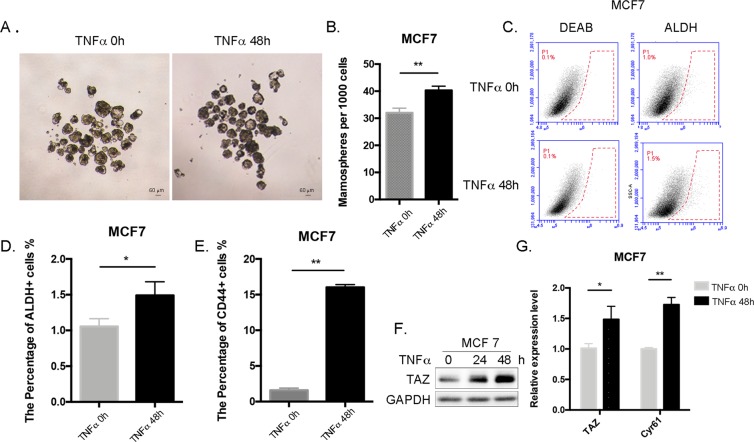
TNF-α increases BCSCs and TAZ expression in MCF-7 breast cancer cells. (**A**) TNF-α increases BCSCs in MCF7 cells, as measured by mammosphere culture. MCF7 cells were treated with 10 ng/ml TNF-α or 0.1% BSA for 48 h, and cells were seeded at a density of 1,000 cells per well and cultured for 14 d. The number of primary mammospheres with diameter ≥60 μm was then quantified. (**B**) The number of primary mammospheres per 1,000 cells initially seeded was quantified (mean ± SEM; n = 3). **P < 0.01, t-test. (**C**) TNF-α increases BCSCs in MCF7 cells as measured by ALDH assays. MCF7 cells were treated with 10 ng/ml TNF-α or 0.1% BSA for 48 h. Cells were collected for aldehyde dehydrogenase assays by FACS. (**D**) The percentage of ALDH + cells was quantified (mean ± SEM; n = 3). *P < 0.05, t-test. (**E**) TNF-α increases BCSCs in MCF7 as measured by CD marker staining. MCF7 cells were treated with 10 ng/ml TNF-α or 0.1% BSA for 48 h. The cells were collected for CD marker staining by FACS. The percentage of CD44 + cells was quantified (mean ± SEM; n = 3). **P < 0.01, t-test. (**F**) TNF-α induces TAZ protein expression in MCF7 cells. MCF7 cells were treated with 10 ng/ml TNF-α or 0.1% BSA for 24 h and 48 h, respectively. TAZ protein levels were measured by Western blotting. (**G**) TNF-α induces *TAZ* and *CYR61* mRNA expression in MCF7 cells. *TAZ* and *CYR61* mRNA levels were analyzed by RT-qPCR. Data are normalized to untreated samples (mean ± SEM; n = 3). **P < 0.01, t-test. NS indicates not significant.

TNF-α is well known as an important effector in breast cancer^36^. To confirm whether TNF-α promotes breast cancer stem-like cells, we cultured MCF7 cells under non-adherent culture conditions to foster mammosphere formation and found that TNF-α addition (10 ng/ml) significantly increased the number of mammospheres (Fig. 1A,B). We further performed ALDEFLUOR assays in MCF7 and MDA-MB-468 cell lines with or without TNF-α treatment and found that TNF-α significantly increased the population of ALDH positive cells in both MCF7 (Fig. 1C,D) and MDA-MB-468 (Fig. S1A,B) cell lines. We also tested the percentage of CD44+ and CD24− cells in these two cell lines. The percentage of CD44+ cells increased significantly in MCF7 (Figs. 1E and S1C). All MCF7 cells are CD24 positive, and all MDA-MB-468 cells are both CD44 and CD24 positive (Fig. S1C,D), as previously reported^37,38^. We found that CD24 was increased in MCF7 cells but decreased in MDA-MB-468 cells after TNF-α treatment (Fig. S1C,D).

Next, we demonstrated that TNF-α promotes protein expression of TAZ, a transcriptional coactivator necessary for self-renewal and tumor initiation in BCSCs^27^ in MCF7 (Fig. 1F). Similar results were observed in MDA-MB-468 cells (Fig. S1E). Because YAP shares a similar regulatory mechanism with TAZ in the Hippo pathway, we tested whether TNF-α also induces YAP. However, YAP was not induced by TNF-α in either MCF7 or MDA-MB-468 cells (Fig. S2C,D).

To characterize the potential mechanism by which TNF-α induces TAZ, we first measured *TAZ* mRNA levels by RT-qPCR. Our results showed that TNF-α significantly up-regulates mRNA levels of both *TAZ* and its target gene *Cyr61* in both MCF7 (Fig. 1G) and MDA-MB-468 (Fig. S1D) cells. We also measured TAZ protein half-life and found that TNF-α does not affect TAZ protein stability (Fig. S2A,B). We concluded that TNF-α up-regulates TAZ expression predominately at the transcriptional level rather than the post-transcriptional level.

### TAZ mediates TNF-α-increased the proportion of BCSCs

**Figure 2:**
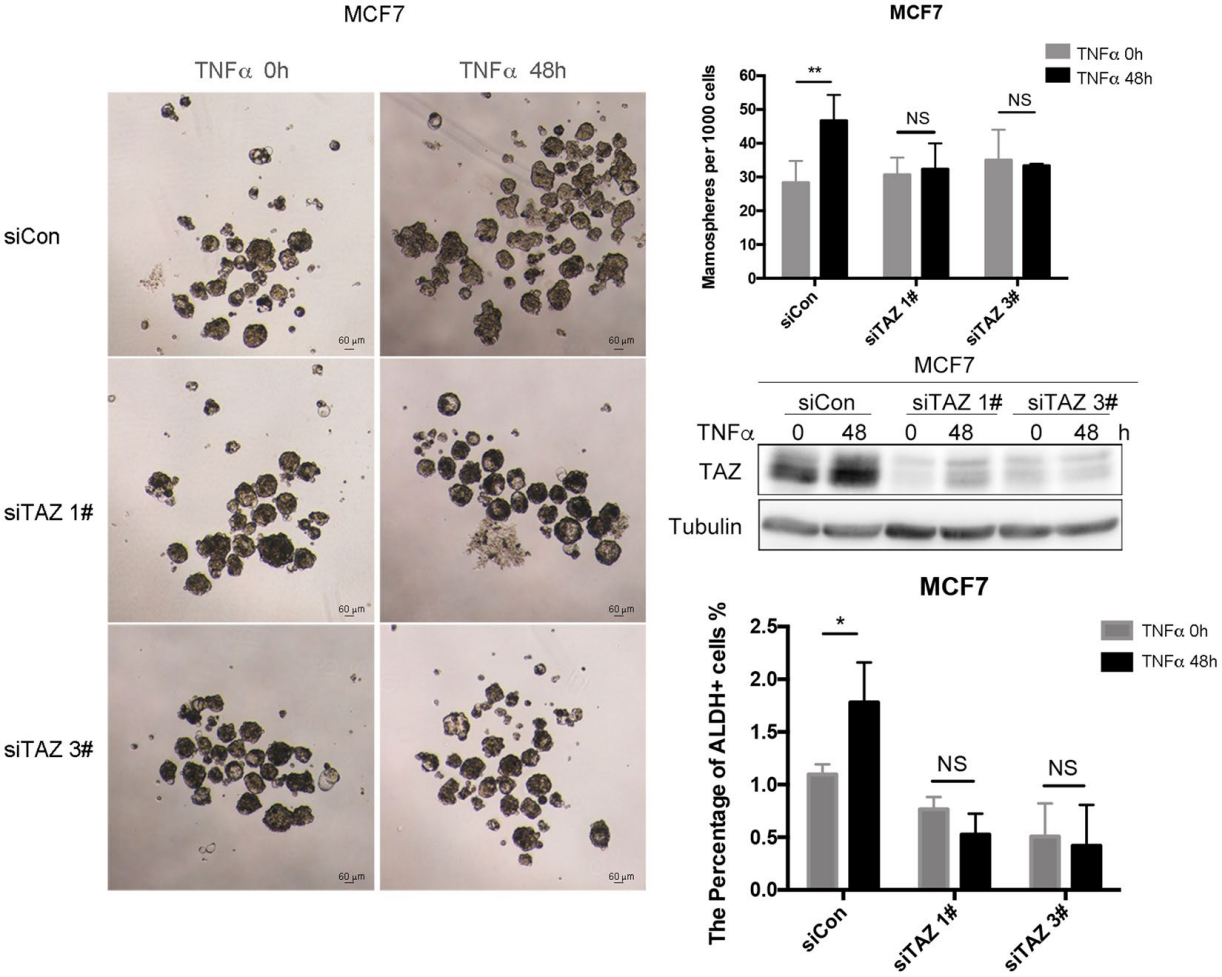
TAZ mediates TNF-α-increased the proportion of breast cancer stem-like cell. (**A**) TAZ depletion blocks TNF-α-promoted BCSC increase, as measured by mammosphere culture. MCF7 cells were transfected with 20 nM TAZ siRNA for 48 h and then exposed to 10 ng/ml TNF-α or 0.1% BSA for 48 h. (**B**) Quantitative data for panel A. **P < 0.01, t-test. NS indicates significant. (**C**) TAZ protein levels were knocked down using siTAZ1# and siTAZ3#. Protein expression was determined by WB. (**D**) TAZ depletion blocks TNF-α-promoted BCSC increase, as measured by ALDH assays. MCF7 cells were transfected with 20 nM TAZ siRNA for 48 h and then exposed to 10 ng/ml TNF-α or 0.1% BSA for 48 h. Cells were collected for ALDH assays by FACS. The percentage of ALDH+ cells was quantified (mean ± SEM; n = 3). *P < 0.05, t-test. NS indicates not significant.

To explore whether TNF-α promotes BCSCs via up-regulation of TAZ, we knocked down TAZ using two individual siRNAs in MCF7 cells and assessed BCSC levels. TNF-α-induced mammosphere increase was completely abolished when TAZ was knocked down (Fig. 2A–C). In agreement with this, TAZ knockdown significantly blocked TNF-α-induced ALDH positive cell increase in MCF7 cells (Fig. 2D). Similar results were observed in MDA-MB-468 cells (Fig. S3A–C). TAZ knockdown also significantly decreased the TNF-α induced increase of CD44+ cells in MCF7 (Fig. S3D,E). TAZ knockdown did not blocked the TNF-α mediated the CD24 expression changes in both cell lines (Fig. S3G). These results indicate that TAZ may be necessary for TNF-α-increased the proportion of BCSCs.

### TNF-α induces TAZ transcription through the non-canonical NF-κB pathway

**Figure 3:**
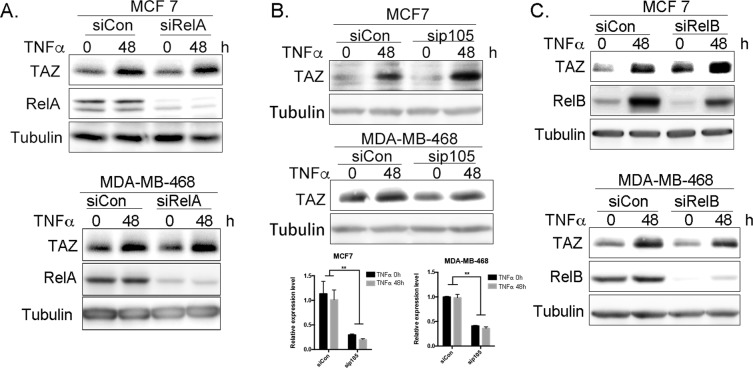
TNF-α induces TAZ not through RelA, RelB, and p105. (**A**) RelA knockdown did not block TNF-α induced TAZ protein expression in both MCF7 and MDA-MB-468. Cells were treated with TNF-α or 0.1% BSA for 48 h and TAZ and RelA proteins were detected by WB. (**B**) p105 knockdown did not block TNF-α induced TAZ protein expression in both MCF7 and MDA-MB-468. Cells were treated with TNF-α or 0.1% BSA for 48 h. The protein level of TAZ were detected by WB. The p105 knockdown was measured by RT-qPCR. (**C**) RelB knockdown did not block TNF-α induced TAZ protein expression in both MCF7 and MDA-MB-468. Cells were treated with TNF-α or 0.1% BSA for 48 h and TAZ and RelB protein was detection by WB.

TNF-α is a well-known activator of the canonical NF-**κ**B pathway, and RelA regulates TAZ transcription in mesenchymal stem cells^32^. To further characterize the mechanism by which TNF-α induces TAZ transcription, we first tested whether RelA is responsible for TNF-α induction of TAZ transcription. After RelA knockdown, TAZ was still induced by TNF-α (Fig. 3A). Next, we knocked down other transcriptional factors in the canonical NF-**κ**B pathway, including p105 and RelB. However, knockdown of neither p105 nor RelB suppressed TAZ induction by TNF-α (Fig. 3B,C). These results indicate that TNF-α may not induce TAZ transcription via the canonical NF-**κ**B pathway.

**Figure 4:**
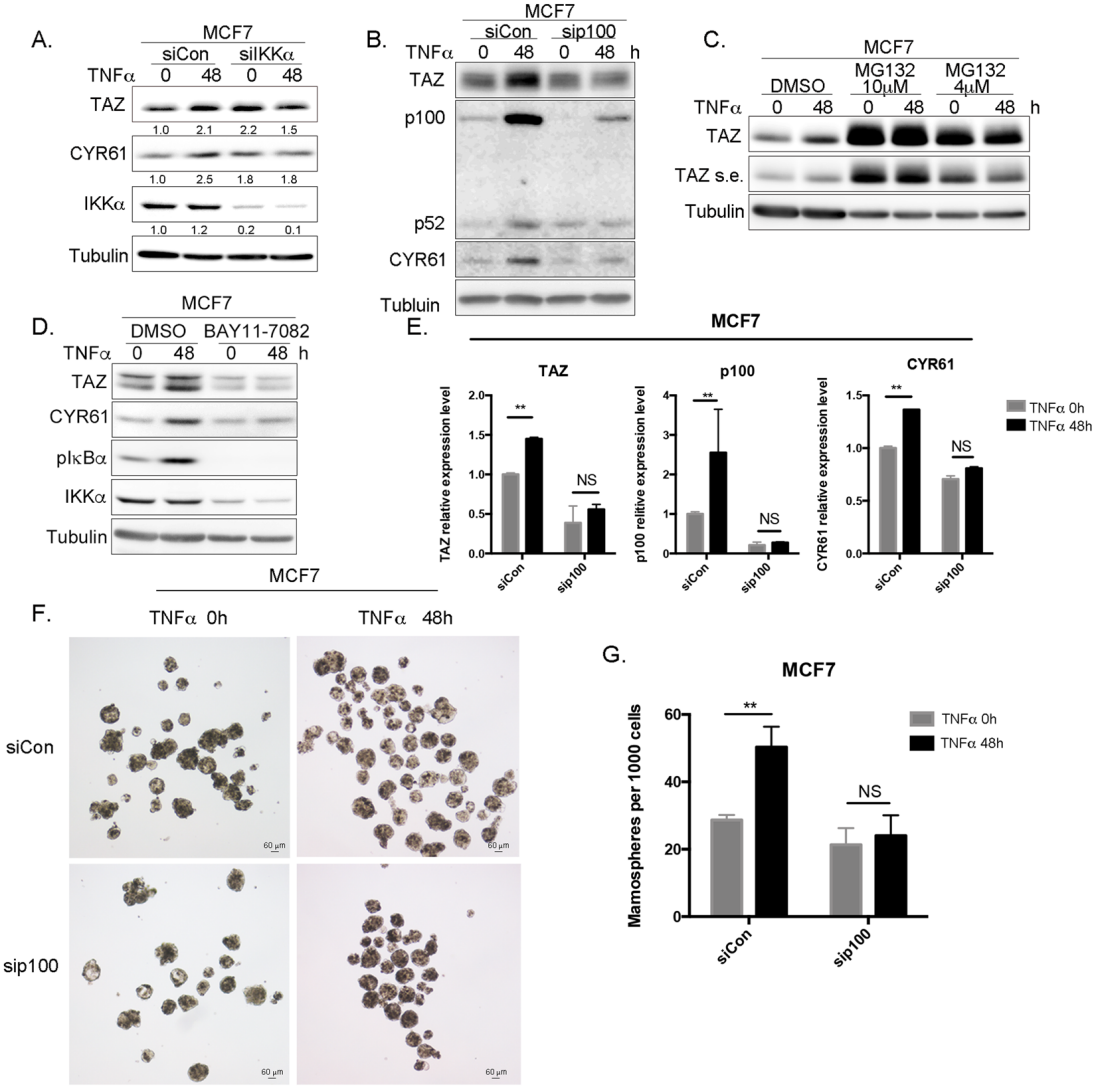
TNF-α induces TAZ through the non-canonical NF-κB pathway. (**A**) IKKα knockdown blocks TNF-α induced TAZ and CYR61 protein expression in MCF7. Cells were treated with TNF-α or BSA for 48 h and TAZ and CYR61 proteins were detected by WB. Band intensities were quantatified by IMAGE J. (**B**) p100 knockdown blocks TNF-α induced TAZ and CYR61 protein expression in MCF7 cells. (**C**) MG132 blocks TNF-α induced TAZ and CYR61 protein expression in MCF7 cells. s.e. indicates the abbreviation of short exposure time. (**D**) BAY11-7082 blocks TNF-α induced TAZ and CYR61 protein expression in MCF7 cells. BAY11-7082 is an NF-κB inhibitor, which inhibits TNF-α and induces IκBα phosphorylation. MCF7 cells were treated with BAY11-7082 (2 μM) and then exposed to TNF-α. (**E**) IKKα knockdown, p100 knockdown, and MG132 block TNF-α induced *TAZ* and *CYR61* mRNA expression in MCF7 cells. Cells were treated with TNF-α or BSA for 48 h. qPCR was performed to detect *TAZ*, *p100*, and *CYR61* mRNA expression levels. **P < 0.01, t-test. (**F**) p100 knockdown blocks TNF-α-induced mammosphere increase in MCF7 cells. Cells were transfected with 20 nM p100 siRNA for 48 h and then exposed to 10 ng/ml or 0.1% BSA TNF-α for 48 h. (**G**) Quantitative data for panel F. **P < 0.01, t-test. p100 knockdown blocks TNF-α-induced p52 protein accumulation in MCF7 cells.

Subsequently, we knocked down IKKα and found that this manipulation suppressed TNF-α-induced TAZ and CYR61 up-regulation (Figs. 4A and S4A). IKKα plays a crucial role in the non-canonical NF-κB pathway. Next, we knocked down transcription factor p100 in MCF7 and MDA-MB-468 cells and found that p100 depletion also inhibited TNF-α-induced TAZ and CYR61 up-regulation at both the protein and mRNA level (Figs. 4B,E and S4B,D). When the non-canonical NF-**κ**B pathway is activated, p100 is processed through the proteasome^39^. We used MG132, a proteasome inhibitor, to pretreat cells before TNF-α stimulation. As expected, MG132 stabilized TAZ protein but suppressed TNFα-induced TAZ up-regulation in both MCF7 and MDA-MB-468 cells (Figs. 4C and S4C). We also pretreated cells with the IKKα inhibitor BAY11-7082 after TNF-α stimulation. Similar to MG132, BAY11-7082 inhibited TNF-α-induced TAZ and CYR61 increase in both MCF7 and MDA-MB-468 cells (Figs. 4D and S4E).Then we preformed ALDEFLUOR assays and found that IKKα silencing suppressed TNF-α-induced ALDH positive cell increase in both MCF7 and MDA-MB-468 cells (Fig. S4F,G). At the same time, we also detected CD44 and CD24 expression levels in these cells. Knockdown of either IKKα or p100 decreased the TNF-α induced increase of CD44+ cells in MCF7 (Fig. S4G,H). In MDA-MB-468 cells, IKKα and p100 knockdown had no significant effect on the expression of CD24 (Fig. S4G,I).

We next silenced p100 in MCF7 cells and found significant suppression of TNF-α-increased the proportion of mammosphere (Fig. 4F–G). Furthermore, TNF-α promoted not only p100 protein expression, but also its processing (Fig. 4B). p100 knockdown suppressed p100 processing into p52, as well as inhibiting TAZ and CYR61 induction by TNF-α (Fig. 4B). These results further support the notion that TNF-α up-regulates TAZ through the non-canonical NF-**κ**B pathway.

### p52 binds to the TAZ promoter to initiate TAZ transcription through TNF-α-induced p100 expression and processing

**Figure 5:**
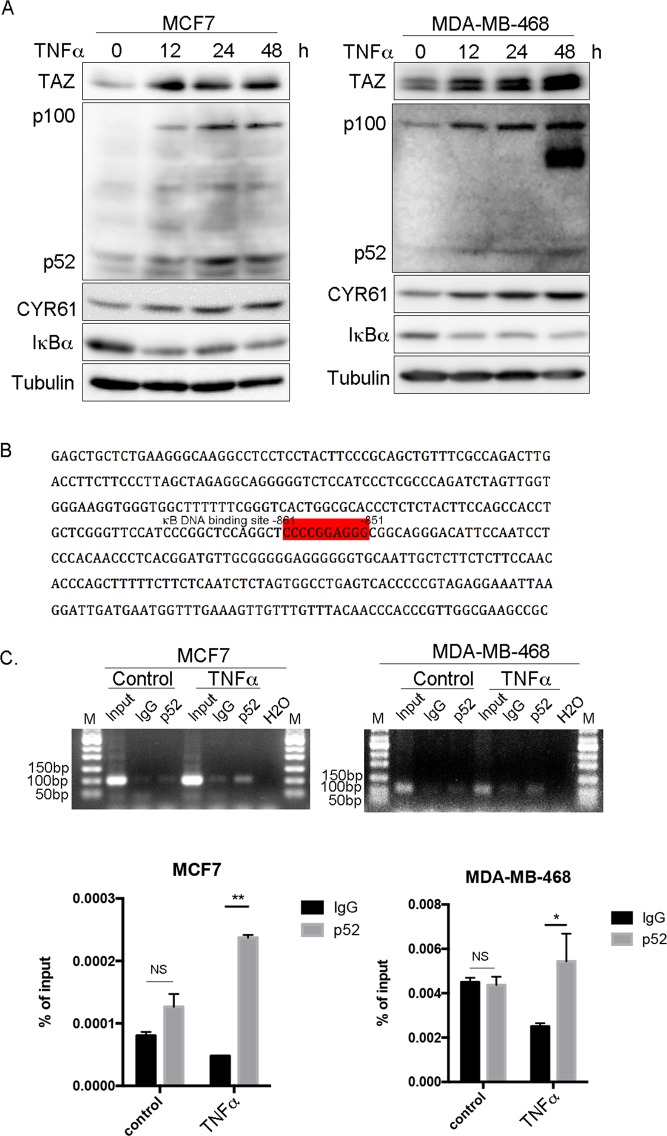
TNF-α induces generation of transcription factor p52 and TAZ transcription. (**A**) TNF-α induces generation of transcription factor p52 from p100 in both MCF7 and MDA-MB-468 cells. Cells were treated with 10 ng/ml TNF-α or 0.1% BSA for 12, 24 and 48 h. TAZ, CYR61 and p100/p52 protein expression levels were determined by WB. (**B**) The *TAZ* promoter sequence contains a p52 binding site, as highlighted in the shaded box. (**C**) TNF-α induces p52 binding to the *TAZ* promoter in both MCF7 and MDA-MB-468 cells, as determined by chromatin Immunoprecipitation (ChIP) assays. Cells were treated with vehicle (PBS) or TNF-α for 3 h. Immunoprecipitation was performed using anti-p52 antibody. PCR or qPCR were performed to quantify precipitated *TAZ* promoter using specific primers flanking the p52 binding site. (**D**).

To further confirm that TNF-α induces TAZ through non-canonical NF-κB signaling, we treated MCF7 and MDA-MB-468 cells with TNF-α and examined p100 processing. We observed that p100 processing was enhanced with time as p52 gradually increased (Fig. 5A). Additionally, we tested whether RANKL, a typical non-canonical NF-κB pathway activator, can induce TAZ. Similar to TNF-α, RANKL also up-regulates TAZ and CYR61 through enhancing p100 processing in MDA-MB-468 cells (Fig. S5A). Furthermore, we performed the ALDEFLUOR assay and found that RANKL significantly increased ALDH positive cells in MDA-MB-468 (Fig. S5B). MCF7 cells are not sensitive to RANKL (100 ng/ml, data not shown).

Then, we tested whether p52 directly promotes TAZ transcription. We cloned the TAZ promoter region (−5000 to ATG) based on the Eukaryotic Promoter Database (http://epd.vital-it.ch/index.php) and searched potential p52 binding sites in the *TAZ* gene promoter region, identifying a p52 binding site from −861 to −851 (Fig. 5B). To determine whether p52 promotes TAZ transcription directly through this site, we performed a chromatin immunoprecipitation (ChIP) assay using an anti-p100/p52 antibody. As expected, the p52 binding site-containing region was immunoprecipitated by the antibody in both MCF7 and MDA-MB-468 cells. Furthermore, p52 binding was increased by TNF-α stimulation (Fig. 5C).

## Discussion

**Figure 6:**
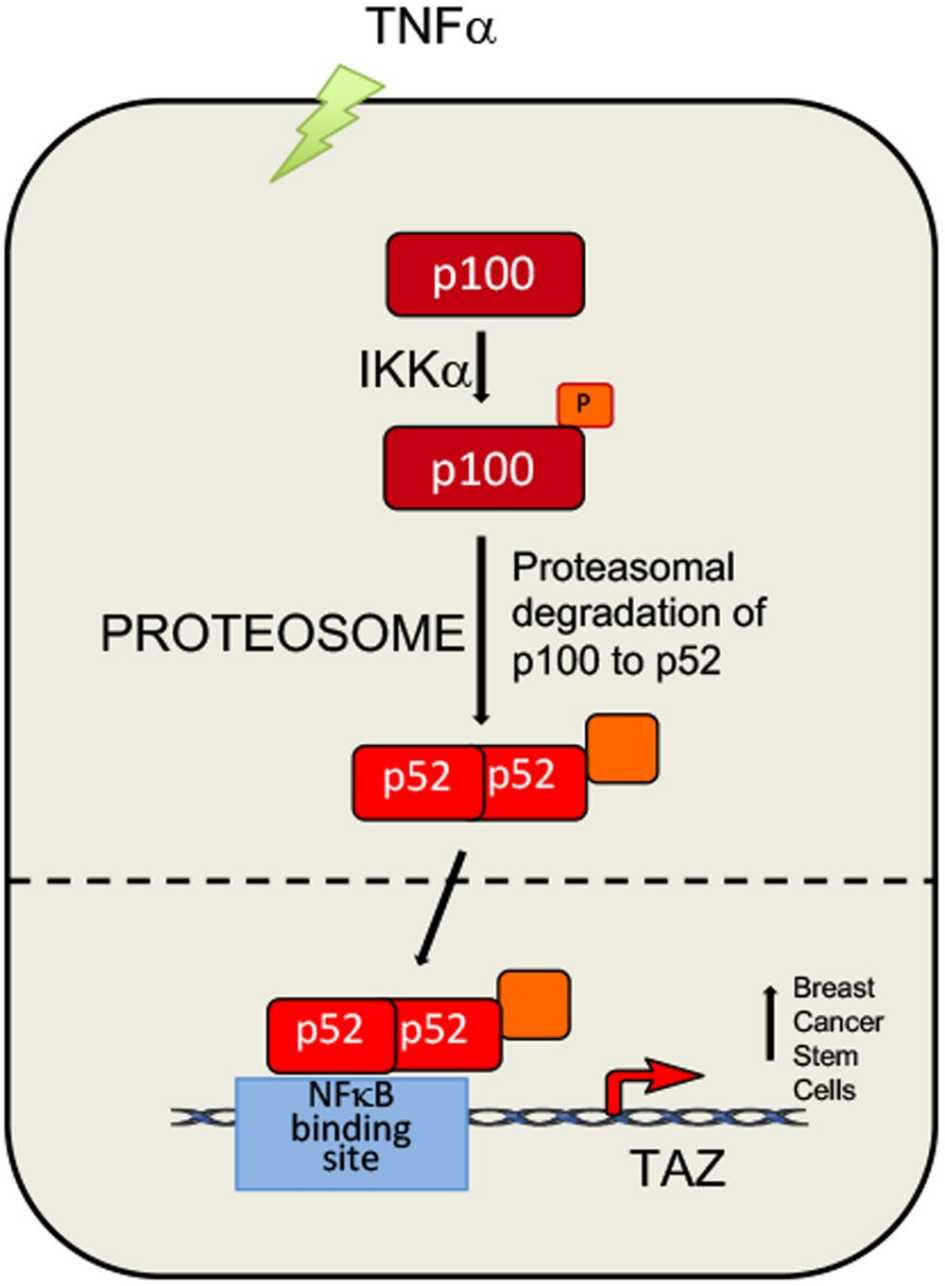
Schematic of the hypothesis that TNF-α increases BCSCs through up-regulation of TAZ expression via the non-canonical NF-κB pathway. TNF-α induces expression of p100 and activates p100 processing into p52 by IKKα and the proteasome. p52 subsequently binds to a p52 binding site within the *TAZ* promoter, subsequently up-regulating the latter’s transcription. TAZ in turn promotes BCSCs in human breast cancer cell lines.

TNF-α triggers immune reaction and promotes the initiation^9,40,41^, proliferation^42,43^, survival^44^, invasion^45,46^ and metastasis of tumor cells. However, the mechanism by which TNF-α promotes BCSCs has not been fully elucidated. In this study, we demonstrate that TNF-α increases BCSCs in MCF7 and MDA-MB-468 breast cancer cell lines through induction of TAZ (but not YAP) transcription. Furthermore, TNF-α induces TAZ expression through the non-canonical NF-κB pathway. Activated p100 is processed into p52 by the proteasome, at which time p52 forms homodimers or heterodimers with another transcriptional factor and translocate to the nucleus, binding to the p52 binding site at the *TAZ* promoter to induce *TAZ* transcription (Fig. 6). Our findings suggest that TAZ plays a crucial role in inflammatory factor TNF-α–increased BCSCs and could serve as a promising therapeutic target.

For the first time, we discovered there is crosstalk between the non-classical NF-κB pathway and the Hippo pathway. Several lines of evidence support that TNF-α induces IKKα-mediated p100 processing into p52, which is translocated to the nucleus and binds to the *TAZ* promoter, inducing its transcription. First, TNF-α induces p100, TAZ, and its downstream target gene CYR61 expression at both the mRNA and protein level. Next, TNF-α increases expression of p52 that is processed from p100 by the proteasome, and knockdown of p100 or administration of the proteasome inhibitor MG132 abrogated induction of TAZ by TNF-α. Furthermore, knockdown of IKKα blocked the induction of TAZ and CYR61 although the exact functional mechanism of IKKα is unknown. On the one hand, IKKα could phosphorylate p100 to activate its procession. On the other hand, IKKα could phosphorylate H3 in the nucleus^47^ to promote TAZ transcription. Finally, RANKL, as a typical non-classical NF-κB pathway activator, also induced expression of TAZ and CYR61.

The correlation between inflammation and cancer has been confirmed by numerous studies. In 1863, Virchow hypothesized the association between cancer and inflammation^48^. To some extent, cancer is a kind of chronic inflammation, and the inflammatory environment supports the development of cancer. In 2008, Frances Balkwill and Alberto Mantovani reported that there were various similarities between tumor and chronic inflammation in the microenvironment^49^. Both immunocytes and inflammatory factors play important roles in tumor development and immunosuppression. TNF-α, a well characterized inflammatory factor, has strong tumor-initiating effects at a low and constant dose. In animal models, blockage of TNF-α and its receptor are strongly antitumor^49^.

It appears that TNF-α increases BCSCs through different mechanisms. In 2010, TNF-α was reported to induce Slug expression by up-regulating HIF1α via the canonical NF-κB pathway, thus promoting BCSCs^18^. In 2012, Li reported that TNF-α up-regulates Twist1 and induces epithelial-mesenchymal transition via the NF-κB pathway^50^. In normal mammary epithelial cells, TNF-α expression levels are very low, whereas TNF-α is over-expressed in most breast tumors^18^. In addition, TNF-α expression levels are significantly positively correlated with recurrence and malignance in these breast cancer patients. In this study under low dose, long-term stimulation, TNF-α increases BCSCs in both MCF7 and MDA-MB-468 cell lines. Importantly, the mechanism of TNF-α-induced BCSCs appears to occur through up-regulation of TAZ expression via the non-canonical NF-κB pathway. TAZ is necessary for TNF-α to increase BCSCs. It has been shown that activated TAZS89A is sufficient to endow CSC-like properties to non-CSCs^27^.

TAZ is an important transcriptional coactivator in the Hippo pathway^18^. High expression levels of TAZ are positively correlated with occurrence and development of cancer^51,52^. TAZ is tightly regulated in response to a wide range of extracellular and intrinsic signals^53^. Estrogen was reported to activate TAZ through G protein-coupled estrogen receptor 1 (GPER, also known as GPR30) and re-organization of F-actin cytoskeleton^54^. Transcriptional regulation of TAZ has not been well studied. It is reported that up-regulation of TAZ expression occurs during osteogenic differentiation of stromal cells derived from human adipose tissue under hypoxic stress conditions^32^. During bone differentiation of human adipose tissue, TNF-α initiates transcription of TAZ by activating the classical NF-κB pathway, which induces p65 translocation to the nucleus, where it binds to the *TAZ* promoter. Under hypoxic conditions, HIF-1 directly binds to the HRE site between the second and third exons of TAZ and promotes *TAZ* mRNA transcription of various breast cancer cell lines^33^. Additionally, the survival rate of breast cancer is negatively correlated with concurrent high-expression of TAZ and HIF-1^33^. Moreover, MRTF/SRF was reported to induce *TAZ* transcription in breast cancer cells in response to Heregulin β1^34^. We found that the p52 transcription factor, part of the non-canonical NF-κB pathway^55^, directly induces *TAZ* transcription in both MCF7 and MDA-MB-468 breast cancer cells in response to TNF-α. Taken together, multiple transcription factors, including HIF-1, SRF, p65, and p52, promote *TAZ* gene transcription in different contexts.

Several questions remain to be addressed in this study. First, since p52 only contains a DNA binding domain but not a transcription activation domain, p52 must be integrated with other factors that have transcriptional activation functionality to form heterodimers. It has been reported that p52 forms heterodimers with RelB or Bcl3 to initiate expression of target genes. In this study, RelB depletion did not influence expression of TAZ induced by TNF-α. Therefore, p52 may activate expression of TAZ by integrating with other distinct transcription factors. Further investigation is required to identify p52 transcriptional factor partners for TAZ induction. In addition, TNF-α significantly induces the expression of p100 in several breast cancer cell lines; however, the molecular mechanism for this is unclear.

In conclusion, we demonstrate that TNF-α launches the cells through a stemness differentiation via up-regulation of TAZ transcription through a non-canonical NF-κB pathway in human breast cancer cell lines. Our findings implicate TAZ as a crucial component in inflammatory factor-promoted breast cancer cells stemness differentiation and suggest that TAZ could serve as a promising therapeutic target in breast cancer.

## Methods

### Cell culture

Human breast cancer cell lines MCF7 and MDA-MB-468 were purchased from American Type Culture Collection (ATCC). These cell lines have been authenticated with STR assays in 2015 by Conservation Genetics CAS Kunming Cell Bank. These cells were cultured in MEM medium supplemented with 10% fetal bovine serum. All cells were maintained at 37 °C in an incubator with 5% CO_2_. The presence of mycoplasma was routinely tested by PCR to eliminate contamination.

### Drugs, reagents and antibodies

TNF-α was purchased from PEPRO TECH (Rocky Hill, USA). RANKL was purchased from R&D Systems (Minneapolis, MN). Cycloheximide (CHX) was purchased from MP Biomedicals (Irvine, CA, USA). BAY 11-7082, LPS and MG132 were purchased from Sigma-Aldrich (St Louis, MO, USA). The anti-TAZ V386 (25937), anti-CYR61 (3491), anti-p100/p52 (4882), anti-RelA (8242) and anti-RelB (4954) antibodies used for WB were purchased from Cell Signaling Technology (Danvers, MA, USA). Anti-tubulin (F7425) and anti-YAP (WH0010413 M1) antibodies were purchased from Sigma-Aldrich. Anti-GAPDH (sc-25778) antibody was purchased from Santa Cruz Biotechnology (Santa Cruz, CA, USA). The anti-p100/p52 (ab7972) antibody used for ChIP assays was purchased from Abcam (Cambridge, MA).

### ALDEFLUOR assay

We performed ALDH assays using an ALDEFLUOR Assay Kit (no. 01700; Stem cell Technologies, Vancouver, BC, Canada) according to the standard protocol. In brief, 25,000 cells were collected and resuspended in 1 ml assay buffer. Next, 5 μl activated reagent was added. Half of the samples (0.5 ml) were immediately put into control tubes with 5 μl DEAB buffer. All samples were incubated for 40 min at 37 °C, after which time they were centrifuged for 5 min at 250 g. Cells were resuspended in 0.5 ml assay buffer and subjected to flow cytometry analysis.

### CD marker staining assays

1 × 10^6^ cells were collected and resuspended in 1 ml 2% fetal bovine serum PBS. Combinations of fluorochrome-conjugated monoclonal antibodies against human CD44 (FITC; cat. #555478) and CD24 (PE; cat. #555428) were obtained from BD Biosciences (San Diego, California, USA). Primary antibodies or the respective isotype controls (BD Biosciences) were added to the cell suspension and incubated at 4 °C in the dark for 30 min. The cells were washed with clod PBS for three times, were resuspended in 0.5 ml 2% fetal bovine serum PBS and were subjected to flow cytometry analysis.

### Mammosphere culture

We performed mammosphere assays using a Mammosphere Culture Kit (no. 05620; Stem cell Technologies). MCF7 cells were first treated with TNF-α for 48 hours and then plated in ultra-low attachment plates (no. 3473, Corning Inc., Corning, NY, USA) at a density of 500 cells per well. The cells were cultured in complete MammoCult™ Medium (Basal Medium + Proliferation Supplement + heparin + hydrocortisone) without TNF-α. Preparation of the complete MammoCult™ Medium was carried out according to the method instructions in Mammosphere Culture Kit (no. 05620; Stem cell Technologies). The number of mammospheres with a diameter >60 μm after 14 days in culture was then quantified.

### Transfection

We used Lipofectamine 2000 (Invitrogen, Carlsbad, CA, USA) for siRNA transfection according to the manufacturer’s recommended protocols. Control siRNA was purchased from RiboBio Co., Ltd. (Guangzhou, Guangdong, China). Other siRNA sequences are listed in Supplementary Table S1.

### RT-qPCR

RNA was extracted using TRIzol reagent (Invitrogen Corp., Carlsbad, CA). Reverse transcription was performed using the iScript cDNA Synthesis Kit (Bio-Rad Laboratories, Hercules, CA), and RNA levels were quantified using SYBR Green Select Mastermix (no. 4472908, Applied Biosystems, Foster, CA, USA) on the ABI-7900HT System (Applied Biosystems). Primer sequences are listed in Supplementary Table S2.

### Chromatin Immunoprecipitation (ChIP)

ChIP was performed using MCF7 and MDA-MB-468 cells, which were cultured in 10-cm dishes and treated with TNF-α (10 ng/ml) for 48 h. Cells were then treated with formaldehyde for crosslinking. Cells were scraped into Eppendorf tubes and centrifuged at 1000 g, 4 °C for 10 min. Cell lysates were sonicated to shear DNA to an average fragment size of 150–500 bp. After sonication, cell debris were removed by centrifugation at 10,000 g at 4 °C for 1 min. DNA-protein complexes were mixed with the antibody-A/G-beads and incubated at 4 °C for 10 h. Chromosomal DNA was purified and analyzed by quantitative PCR. Primers for the *TAZ* gene promoter p52 binding site were as follows: 5′-TCTACTTCCAGCCACCTGC-3′ (forward) and 5′-GCAACATCCGTGAGGGTTG-3′ (reverse).

## Supplementary information

Supplementary Information
