# Supplementary material for: TNF-α increases breast cancer stem-like cells through up-regulating TAZ expression via the non-canonical NF-κB pathway
Source: Sci Rep. 2020 Feb 4;10:1804. doi: 10.1038/s41598-020-58642-y (PMC7000832; doi:10.1038/s41598-020-58642-y)
Supplement: Supplementary file 1 — Supplementary Information [file 41598_2020_58642_MOESM1_ESM.pdf]

TNF-α increases breast cancer stem-like cells through up-regulating TAZ expression via the non-canonical NF-κB pathway

Wenjing Liu[1, 2, 3#], Xiaoqing Lu[4#], Peiguo Shi[1#], Guangxi Yang[1], Zhongmei Zhou[1], Wei Li[5,3], Xiaoyun Mao[6*], Dewei Jiang[1*], Ceshi Chen[1, 7*]

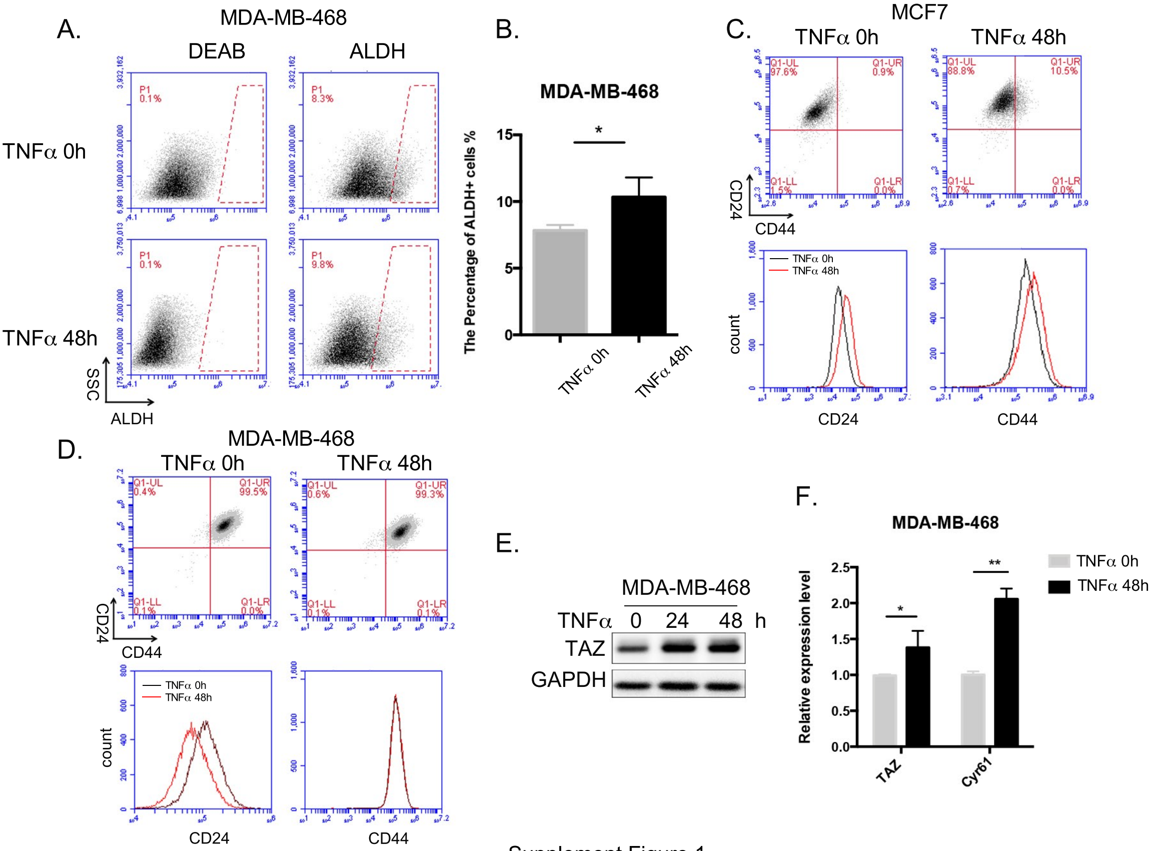

Supplement Figure 1

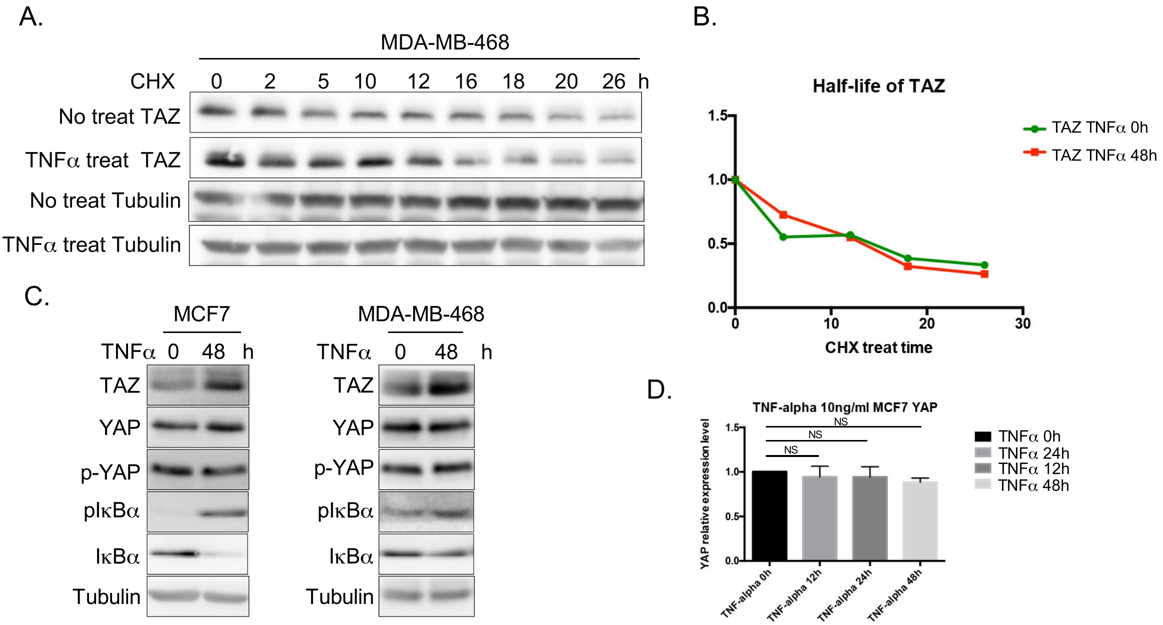

Supplement Figure 2

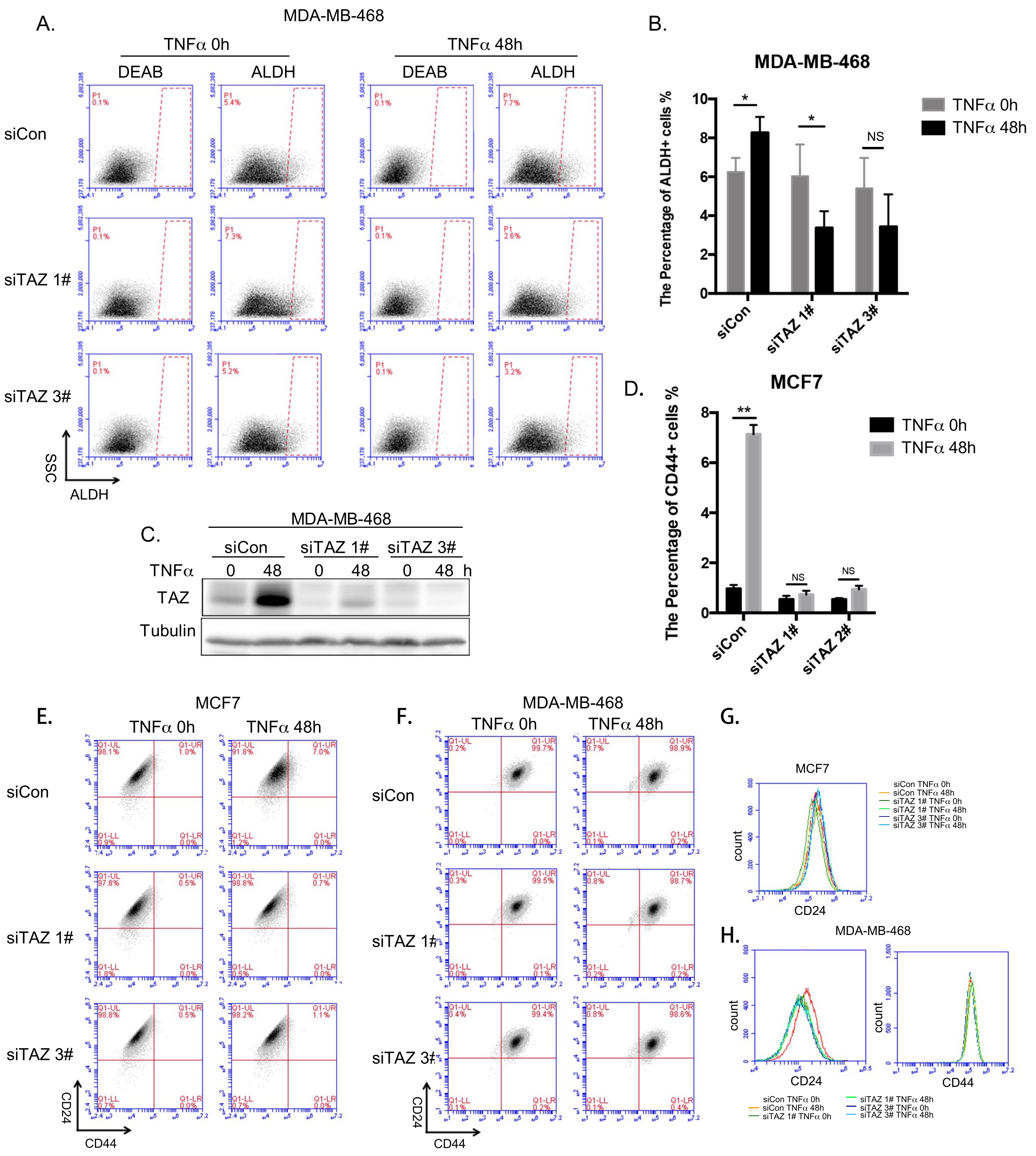

Supplement Figure 3

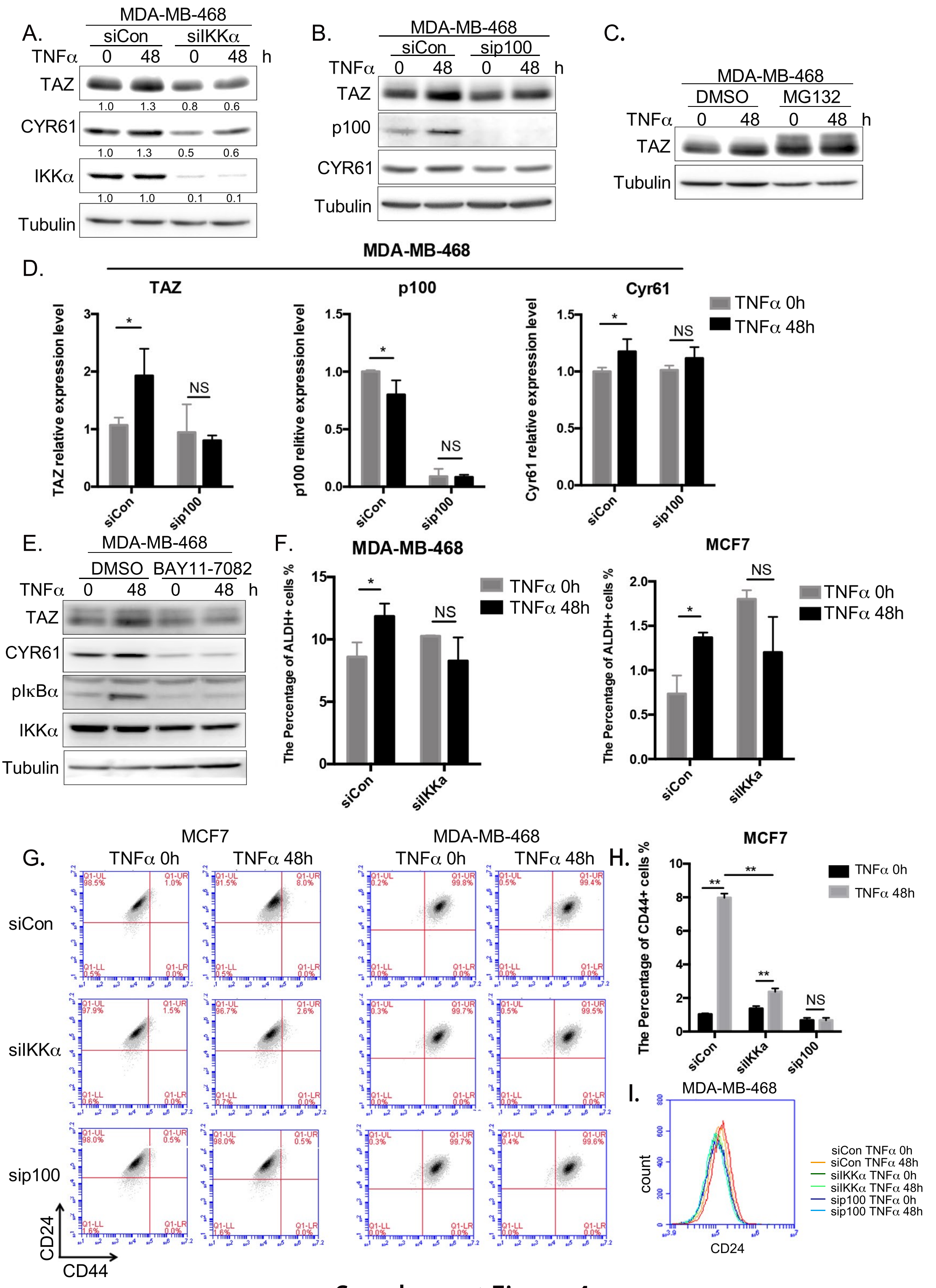

Supplement Figure 4

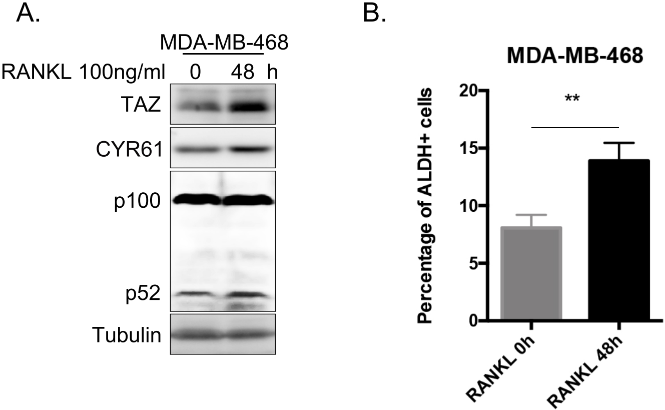

Supplement Figure 5

Supplementary Figure Legends

Figure. S1 TNF-α increases BCSCs and TAZ expression in MDA-MB-468 breast cancer cell line.

A. TNF-α increases BCSCs as measured by ALDH assays. MDA-MB-468 cells were treated with 10 ng/ml TNF-α or 0.1% BSA for 48 h. The cells were collected for aldehyde dehydrogenase assays by FACS.

B. The percentage of ALDH+ cells was quantified (mean ± SEM; n = 3), *P<0.05, t-test.

C. TNF-α increases BCSCs in MCF7 as measured by CD44 and CD24 staining. MCF7 cells were treated with 10 ng/ml TNF-α or 0.1 % BSA for 48 h. The cells were collected and subjected for staining and FACS analysis.

D. TNF-α increases BCSCs in MDA-MB-468 as measured by CD44 and CD24 staining. MDA-MB-468 cells were treated with 10 ng/ml TNF-α or 0.1 % BSA for 48 h. The cells were collected and subjected for staining and FACS analysis.

E. TNF-α induced TAZ protein expression. MDA-MB-468 cells were treated with 10 ng/ml TNF-α or 0.1% BSA for 24 h and 48 h, respectively. The TAZ protein levels were measured by WB.

F. TNF-α induced *TAZ* and *Cyr61* mRNA expression. MDA-MB-468 cells were treated with 10 ng/ml TNF-α or 0.1% BSA for 48 h, *TAZ* and *CYR61* mRNA levels were analyzed by RT-qPCR. The data were normalized to untreated samples (mean ± SEM; n = 3). *P<0.05, **P<0.01, t-test.

Figure. S2 TNF-α does not affect TAZ protein stability

A. TNF-α did not extend TAZ protein's half-life in MDA-MBA-468 cells, as detected by CHX chase assays.

B. Quantitative data for panel A.

C. TNF-α did not induce YAP protein expression in MCF7 and MDA-MB-468 cells. The cells were treated with 10 ng/ml TNF-α or 0.1% BSA for 48 h and the protein levels of TAZ and YAP were detected by Western blotting.

D. TNF-α did not induce *YAP* mRNA expression in MCF7. The cells were treated with 10 ng/ml TNF-α or 0.1% BSA for 12 h, 24 h, and 48 h especially, and *YAP* mRNA levels were analyzed by RT-qPCR. Data are normalized to untreated sample (mean ± SEM; n = 3). **P<0.05, P<0.01, t-test.

Figure S3. TAZ mediates TNF-α-induced BCSC increase in MDA-MB-468.

A. TAZ depletion blocked TNF-α-induced BCSC increase, as measured by ALDH assays. MDA-MB-468 cells were transfected with 20 nM TAZ siRNA for 48 h and then exposed to 10 ng/ml TNF-α or 0.1% BSA for 48 h. The cells were collected for ALDH assays by FACS.

B. Quantitative data for panel A (mean ± SEM; n = 3). *P<0.05, t-test.

C. TAZ proteins were knocked down by siTAZ1# and siTAZ3#. The protein expression was determined by WB.

D. Knockdown TAZ decreased the percentage of CD44+ cell increase in response to TNF-α in MCF7. MCF7 cells were transfected with 20 nM TAZ siRNA for 48 h and then treated with 10 ng/ml TNF-α or 0.1% BSA for 48 h. The cells were collected for CD marker staining by FACS. The percentage of CD44+ cells was quantified (mean ± SEM; n = 3). **P<0.01, t-test.
E. The FACS histogram of panel D.
F. The expression changes of CD44 and CD24 in MDA-MB-468 cells after TAZ knockdown. The cells were transfected with 20 nM TAZ siRNA for 48 h and then treated with 10 ng/ml TNF-α or 0.1% BSA for 48 h. The cells were collected for staining and FACS analysis.
G. The quantitative results of CD24 in panel E.
H. The quantitative results of CD44 and CD24 in panel F.

Figure S4. TNF-α induces TAZ through non-canonical NF-κB pathway.
A. IKKα knockdown blocked TNF-α induced TAZ and CYR61 protein expression in MDA-MB-468. The cells were treated with TNF-α for 48 h and TAZ and CYR61 proteins were detection by WB.
B. p100 knockdown blocked TNF-α induced TAZ and CYR61 protein expression in MDA-MB-468. The cells were treated with TNF-α or BSA for 48 h and TAZ, p100 and CYR61 proteins were detection by WB.
C. MG132 blocked TNF-α induced TAZ and CYR61 protein expression in MDA-MB-468 cells.
D. p100 knockdown blocked TNF-α induced *TAZ* and *CYR61* mRNA expression in MDA-MB-468 cells. The cells were treated with TNF-α for 48 h. qPCR was performed to detect *TAZ*, *p100*, and *CYR61* mRNA expression levels. *P<0.05, t-test.
E. BAY11-7082 blocked TNF-α induced TAZ and CYR61 protein expression in MDA-MB-468. MDA-MB-468 cells were treated with BAY11-7082 (4 μM) or DMSO and then exposed to TNF-α or BSA. TAZ and CYR61 protein levels were detected by WB.
F. IKKα depletion blocked TNF-α-induced BCSC increase, as measured by ALDH assays. MCF7 and MDA-MB-468 cells were transfected with 20 nM IKKα siRNA for 48 h and then exposed to 10 ng/ml TNF-α or 0.1% BSA for 48 h. The cells were collected for ALDH assays by FACS. The percentage of ALDH+ cells was quantified (mean ± SEM; n = 3). *P<0.05, t-test.
G. The expression changes of CD44 and CD24 in MCF7 and MDA-MB-468 cells after IKKα or p100 knockdown. MCF7 or MDA-MB-468 cells were transfected with 20 nM IKKα or p100 siRNA for 48 h and then treated with 10 ng/ml TNF-α or 0.1% BSA for 48 h. The cells were collected for staining and FACS analysis.
H. The percentage of CD44+ MCF7 cells was quantified in panel G (mean ± SEM; n = 3). **P<0.01, t-test.
I. The CD24 expression changes of MDA-MB-468 cells in panel G.

Figure. S5 RANKL, a typical non-canonical NF-κB pathway activator, induced TAZ and BCSCs in MDA-MB-468 cells.

A. RANKL induced p52, TAZ and CYR61 protein level in MDA-MB-468. MDA-MB-468 cells were treated with 100 ng/ml RANKL or 0.1% BSA for 48 h. TAZ, CYR61 and p100/p52 protein levels were detected by WB.

B. MDA-MB-468 cells were treated with 100 ng/ml RANKL or 0.1% BSA for 48 h. RANKL significantly increased the percentage of BCSCs in MDA-MB-468, as measured by ALDH assays. The percentages of ALDH+ cells were quantified（mean ± SEM; n = 3）. **P<0.01, t-test.

Supplementary Tables

Table S1. siRNA sequences used in this study

Target gene	**（5'→3'）**
TAZ 1#	AAACACCCAUGAACAUCAA
TAZ 3#	AGAGGTACTTCCTCAATCA
IKKa	GCAGGCUCUUUCAGGGACA
p100	GCTGCTAAATGCTGCTCAGAA
RelA（p65）	GCCCUAUCCCUUUACGUCA
RelB	GCCCGUCUAUGACAAGAAA
p105	CCTTCCGCAAACTCAGCTTTA

Table S2. Primer sequences used in this study

**Genes**	**Primer sequences（5'→3'）**	
TAZ	Forward	GGCTGGGAGATGACCTTCAC
	Reverse	CTGAGTGGGGTGGTTCTGCT
YAP	Forward	TAGCCCTGCGTAGCCAGTTA
	Reverse	TCATGCTTAGTCCACTGTCTG
Cyr61	Forward	AGCCTCGCATCCTATACAACC
	Reverse	TTCTTTCACAAGGCGGCACTC
p100	Forward	TGCCATTGTGTTCCGGACA
	Reverse	TGTTTGGAATCAGACACGTCCC
